# Cognitive functioning in adolescents with migraine

**DOI:** 10.1590/S1980-57642016DN10100009

**Published:** 2016

**Authors:** Melissa Andréia Costa-Silva, Ana Carolina de Almeida Prado, Leonardo Cruz de Souza, Rodrigo Santiago Gomez, Antônio Lúcio Teixeira

**Affiliations:** 1 Headache Clinic, University Hospital, Federal University of Minas Gerais, Belo Horizonte, Brazil; 2 Neuroscience Division, Interdisciplinary Laboratory of Medical Investigation, School of Medicine, Federal University of Minas Gerais, Belo Horizonte, Brazil

**Keywords:** migraine, neuropsychology, adolescents, cognitive dysfunction, neuropsychological assessment, migrânea, neuropsicologia, adolescentes, disfunção cognitiva, avaliação neuropsicológica

## Abstract

Although migraine is highly prevalent in children and teenagers, it often goes undetected in these patients, resulting in underdiagnosis and inadequate treatment. Several studies have investigated cognitive changes in adults with migraine. However, there are few studies focusing on children and adolescents.

**Objective:**

To investigate cognitive performance of adolescents with migraine.

**Methods:**

Twenty-eight adolescents diagnosed with migraine and twenty-six individuals without a history of headache were recruited for the study. All participants were evaluated using standardized neuropsychological tests.

**Results:**

Adolescents with migraine had worse performance on tests evaluating short- and long-term verbal memory, attention, executive function, and speed of processing information than controls.

**Conclusion:**

Cognitive dysfunction is common in adolescents with migraine. Since the cognitive deficits found in adolescents with migraine are similar to those reported in adults with migraine, cognitive impairment seems to persist throughout life.

## RESUMO

Apesar de apresentar uma prevalência alta em crianças e adolescentes, nem sempre a migrânea é corretamente identificada nessa faixa etária, o que resulta em tratamentos inadequados e ineficazes. Vários estudos investigaram disfunções cognitivas associadas à migrânea em adultos. No entanto, poucos foram direcionados às crianças e aos adolescentes.

**Objetivo:**

Investigar o funcionamento cognitivo de adolescentes com migrânea.

**Métodos:**

Participaram do estudo 28 adolescentes diagnosticados com migrânea e 26 controles. Os participantes foram avaliados através de testes neuropsicológicos padronizados.

**Resultados:**

Os adolescentes com migrânea apresentaram pior desempenho que controles em testes que avaliaram memória verbal de curto e de longo prazo, atenção, funções executivas, velocidade de processamento.

**Conclusão:**

Disfunção cognitiva é comum em adolescentes com migrânea. Os resultados apontam para a persistência dos prejuízos cognitivos ao longo da vida, uma vez que os prejuízos encontrados nos adolescentes são semelhantes aos encontrados em adultos com migrânea.

## INTRODUCTION

Headache is a symptom often present during childhood and adolescence, and one of the most frequent complaints in pediatric offices. Despite this, the condition is often underdiagnosed, resulting in inadequate treatment.^1^ Migraine is highly prevalent among children and adolescents, affecting approximately 10% of all teenagers aged between 10- and 18.^1^^-^ 3

Several studies have indicated cognitive changes in patients with migraine.^4^^-^ 9 There is evidence that adults with migraine may present deficits in attention, processing speed (reaction time), verbal memory, working memory, and verbal skills.^4^^-^ 9 Regarding other cognitive domains, such as visual memory and visuospatial/constructional skills, results are more conflicting.^5^ Few studies have assessed the impact of migraine parameters, such as episode frequency and duration, on cognitive performance, and results have failed to show any significant influence of these factors.^4^^-^ 6 ^,^ 10 ^-^ 12

Scant information is available regarding the impact of migraine on the cognition of children and adolescents.^4^ A previous study involving children with migraine aged 7 to 11 years found no global intelligence or visual-motor skill deficits, but identified short and long-term memory impairments.^13^ Inhibition deficits, impairment in speed of processing information, and in selecting and shifting attention were also reported in children suffering from migraine.^14^ These findings may partly explain the complaints related to poor academic performance in migraine subjects.^15^

Considering that childhood and adolescence are pivotal periods for appropriate schooling, and that cognitive deficits related to migraine may interfere with academic performance, thorough investigation of cognitive impairment in migraine subjects is crucial for subsequent follow-up by physicians.

The goals of the present study were: [1] to compare the cognitive performance of children and adolescents with migraine against performance of subjects without neurological disorders; and [2] to identify the neuropsychological tests which are sensitive to the cognitive alterations associated with migraine.

## METHODS

**Subjects.** Fifty-four subjects aged 10 to 18 years were recruited for this study. Twenty-eight patients (22 women, 6 men) diagnosed with migraine (according to the Headache Society criteria)^16^ attended the headache service at the Neurology Service of the Federal University of Minas Gerais. Twenty-six individuals (19 women, 7 men) without a history of headache episodes were recruited from friends and family members of patients to ensure socioeconomic status matching. 

Exclusion criteria for all subjects were as follows: [1] presence of headache, except migraine in the patient group; [2] history of head trauma; [3] history of any psychiatric disorder; [4] and history of other clinical conditions (concomitant disease). All subjects were examined by both a neurologist and a psychiatrist who also assessed participants' ability to understand the cognitive tests. If eligible, patients and controls were subjected to neuropsychological evaluation. All patients were free of pain and migraine effects during the neuropsychological evaluation session. No patients were in use of prophylactic medication. 

**Instruments.** Neuropsychological evaluation was perf ormed by a trained neuropsychologist (MACS). Episodic memory and learning were evaluated by the Rey Auditory Verbal Learning Test (RAVLT). The Trail-Making Test (A and B) (TMTa and TMTb) and the Stroop Test (ST) were used to evaluate executive functions. The Verbal Fluency Test (VFT) was administrated to assess language abilities. These instruments are traditional neuropsychological tests and have been widely used in neuropsychological assessment of children and adolescents with migraine.^10^^-^ 14

All tests were administrated within a single session. Informed consent was obtained from children and adolescents' parents or guardians. The study was approved by the Ethics Committee of the Federal University of Minas Gerais (COEP/UFMG).

**Statistical analysis.** Variables were checked for normality with the Kolmogorov-Smirnov test. For statistical analyses, the Chi-Square test was employed to compare categorical variables. As continuous data have a parametric distribution, Student's t-tests were used to compare control vs. migraine groups. The significance level was < 0.05. All statistical analyses were performed using the Statistical Package for Social Sciences (SPPSS version 11.5).

## RESULTS

There were no differences in age, sex and education between groups (Table 1).

**Table 1.** Descriptive data.

	**Migraineurs**	**Controls**	**p**		
Sample size	28	26			
Gender (% female)	78.57	73.07	0.75		
	**Mean**	**SD**	**Mean**	**SD**	**p**
Age (years)	14.39	2.6	14.58	2.43	0.79
Education (years)	7.79	2.42	7.88	2.28	0.87

Neuropsychological assessment showed significant differences between groups in short-term and long-term verbal memory (RAVLT), attention and executive functions (ST and TMT) and speed of processing information and visuomotor tracking and selective attention (TMTa and TMTb) (Table 2). Subjects with migraine performed worse than controls on all tests.

**Table 2.** Performance on neuropsychological tests

**Test**		**Controls Mean (SD)**	**Migraineurs Mean (SD)**	**p**
RAVLT	A1*	7.19 (1.93)	6.00 (1.41)	0.012
	A2*	10.62 (1.79)	9.14 (2.30)	0.012
	A3*	12.50 (1.27)	10.89 (2.00)	0.001
	A4*	12.96 (1.53)	11.64 (2.14)	0.013
	A5*	13.92 (1.54)	12.11 (2.06)	0.001
	S A1A5*	57.19 (6.24)	49.79 (8.48)	0.001
	B1*	7.04 (1.21)	5.93 (1.78)	0.011
	A6*	12.69 (2.18)	10.14 (2.41)	0.000
	A7*	13.04 (1.68)	9.79 (2.94)	0.000
	Recognition*	14.62 (0.57)	13.79 (1.75)	0.025
Trail-Making Test	Trail A (time, s)*	30.50 (8.08)	40.04 (18.65)	0.02
	Trail A (errors)	0.62 (0.94)	0.32 (0.61)	0.176
	Trail B (time, s)*	77.19 (34.72)	110.36 (68.89)	0.032
	Trail B (errors)	0.88 (1.24)	1.21 (1.28)	0.344
Stroop Test	Dots (time, s)*	14.19 (2.59)	16.04 (2.84)	0.016
	Dots (errors)	0.54 (0.81)	0.68 (0.90)	0.553
	Words (time, s)	17.31 (3.96)	19.14 (4.34)	0.112
	Words (errors)	0.31 (0.47)	0.32 (0.72)	0.935
	Colors (time, s)	26.31 (4.96)	29.29 (7.43)	0.092
	Colors (errors)	1.85 (1.78)	1.32 (1.41)	0.235
Verbal Fluency Test	F	11.69 (3.69)	11.14 (2.82)	0.54
	A	10.08 (3.68)	9.39 (2.98)	0.45
	S	9.58 (3.26)	9.64 (3.12)	0.94
	S FAS	31.38 (9.12)	30.14 (7.64)	0.589
	Animals*	18.15 (4.79)	14.71 (4.36)	0.008

*p<.05

Regarding RAVLT, migraine patients had significantly lower scores than control subjects. This result indicates changes in verbal memory and cued recall and recognition of verbal stimuli. Significant differences were detected in visuomotor selective attention and speed of processing information between groups, as measured by reaction time (time in seconds) on the TMTa and TMTb. On the TMT, there were no differences in error rates between groups. Migraineurs also scored significantly lower on the ST than controls. There were no significant differences in word reading or ink color naming between groups. Lastly, regarding the VFT, patients with migraine displayed lower performance in the animals category. There were no significant differences in phonemic verbal fluency (FAS test).

## DISCUSSION

Patients with migraine had poorer cognitive performance on neuropsychological testing than controls. This result supports previous findings showing that children and adolescents with migraine have impaired short- and long-term verbal memory, speed of processing information, and selective and divided attention.^13^^,^ 14 ^,^ 17 ^,^ 18

We observed that teenagers with migraine had verbal memory and learning impairments. Migraineurs are more affected by distractors during the learning process and have difficulties in recognition and recall. The poor test performance of migraineurs suggests deficits during registration, consolidation, and recall of verbal stimuli. These deficits are associated with changes in the ability to organize thoughts and to use strategies to search for information. The impairment in short- and long-term memory in children with migraine may be linked to learning difficulties.^13^

We found significant differences between groups for execution time on the TMT where migraineurs took longer to complete the test. Similar results have previously been reported.^14^ The TMT measures executive functioning and is associated with selective and divided attention, speed of processing information, and visuomotor tracking. Deficits in visuomotor tracking and selective attention are consistent with the available data on adults^8 ^and children^17^ with migraine, but one study found no significant differences in sequential and simultaneous information processing between children with migraine and controls.^19^ Calandre et al.^8^ found differences only in reaction time between migraineurs and controls. To reconcile these findings, it is tempting to hypothesize that the speed of information processing might be the first sign of cognitive dysfunction in migraine. Over time, other cognitive domains may be progressively compromised. Interestingly, visuomotor processing speed has been reported to be one of the main deficits associated with white matter abnormalities. These are frequent unspecific findings described in brain magnetic resonance imaging studies of patients with migraine.^8^

The neurochemical and molecular *substrates related to cognitive alterations in migraine a*re *not yet* fully understood. Neuroimaging studies have found associations between white matter abnormalities and headache frequency in adults.^20^^-^ 22 Moreover, neurotransmitters known to be involved with cognition such as dopamine, noradrenaline, and glutamate, seem to play a key role in the pathophysiology of migraine.^23^

Studies evaluating the verbal and phonological (F, A and S) fluency of adults reported no changes in cognitive function.^7^^,^ 10 ^,^ 24 The current study found no significant difference in verbal fluency performance between patients and controls. However, the migraine group showed poorer semantic verbal fluency performance (animals). There are no consistent data in the literature on semantic verbal fluency in adolescents with migraine. Quantitative disparities found in semantic and phonological verbal fluency may suggest that the two groups have significant differences in long-term memory representation and strategies used during the retrieval process. Semantic fluency seems to depend more on access to semantic memory and its integrity than on executive processes, with greater temporal lobe activation. In phonological fluency, the process of searching requires the creation of non-habitual strategies based on lexical representation, also with greater frontal lobe activation.^25^^,^ 26

In a cohort study assessing cognitive and academic performance in patients aged 3 to 26, subjects with migraine were slightly, but significantly impaired in comparison to individuals with tension type headache and headache-free controls.^18^ The tests used in this study measured verbal ability (especially language reception) in patients aging from 3 to 13 years, regardless of the existence of any headache history, medication use, severity, or duration of episode. The association between migraine and verbal functioning also appeared to impact later academic performance.

The current study has several limitations, including its cross-sectional design and small sample size. The restrictive study inclusion criteria, i.e. lack of any psychiatric disorder and not to be under prophylactic treatment, partly explain the study numbers, and reflect the challenges of recruiting such patients. The study is clinic-based where patients were drawn from a referral center where more severe or disabling cases are usually followed. Therefore, the current findings must be viewed with caution as they do not necessarily reflect the cognitive status of patients in the community or individuals with milder forms of migraine.^5^^,^ 6 Also, the aforementioned findings have frequently been observed in other neurological conditions and do not seem to be specific to migraine. Future studies involving larger samples and enrollment of subjects from the community are warranted to overcome these limitations. In future studies, it is of paramount importance to evaluate whether cognitive changes identified impact school performance and/or academic achievement. 

In conclusion, the current results suggest that children and teenagers with migraine may exhibit cognitive symptoms. As similar changes are reported in adult patients with migraine,^8^^,^ 27 ^,^ 28 it remains to be established whether cognitive impairment persists throughout life and/or re-emerges later.

## References

[1] Lagman-Bartolome AM, Lay C (2015). Pediatric migraine variants: a review of epidemiology, diagnosis,
treatment, and outcome. Curr Neurol Neurosci Rep.

[2] Wöber-Bingöl C (2013). Epidemiology of migraine and headache in children and
adolescents. Curr Pain Headache Rep.

[3] Barea L, Tannhauser M, Rotta N (1996). An epidemiologic study of headache among children and adolescents of
southern Brazil. Cephalalgia.

[4] Araújo CM, Barbosa IG, Lemos S, Domingues RB, Teixeira AL (2012). Cognitive impairment in migraine. Dement Neuropsychol.

[5] Suhr JA, Seng EK (2012). Neuropsychological functioning in migraine: clinical and research
implications. Cephalalgia.

[6] Rist PM, Kurth T (2013). Migraine and cognitive decline: a topical review. Headache.

[7] Le Pira F, Zappala G, Giuffrida S (2000). Memory disturbances in migraine with and without aura: a strategy
problem?. Cephalalgia.

[8] Calandre E, Bembibre J, Arnedo M, Becerra D (2002). Cognitive disturbances and regional cerebral blood flow abnormalities
in migraine patients: their relationship with the clinical manifestations of the
illness. Cephalalgia.

[9] Schmitz N, Arkink E, Mulder M (2008). Frontal lobe structure and executive function in migraine
patients. Neurosci Lett.

[10] Le Pira F, Lanaia F, Zappala G (2004). Relationship between clinical variables and cognitive performances in
migraineurs with and without aura. Funct Neurol.

[11] Leijdekkers M, Passchier J, Goudswaard P, Menges L, Orlebeke J (1990). Migraine patients cognitively impaired. Headache.

[12] Pearson A, Chronicle E, Maylor E, Bruce L (2006). Cognitive function is not impaired in people with a long history of
migraine: a blinded study. Cephalalgia.

[13] D'Andrea G, Nertempi P, Milone F, Joseph R, Cananzi A (1989). Personality and memory in childhood migraine. Cephalalgia.

[14] Villa T, Correa Moutran A, Sobirai Diaz L (2009). Visual attention in children with migraine: a controlled comparative
study. Cephalalgia.

[15] Rocha-Filho PA, Santos PV (2014). Headaches, quality of life, and academic performance in schoolchildren
and adolescents. Headache.

[16] Headache Classification Subcommittee of the International Headache
Society (2004). The international classification of headache disorders. Cephalalgia.

[17] Riva D, Aggio F, Vago C (2006). Cognitive and behavioral effects of migraine in childhood and
adolescence. Cephalalgia.

[18] Waldie K, Hausmann M, Milne B, Poulton R (2002). Migraine and cognitive function: A life-course study. Neurology.

[19] Haverkamp F, Honscheid A, Muller-Sinik K (2002). Cognitive development in children with migraine and their healthy
unaffected siblings. Headache.

[20] O'Bryant S, Marcus D, Rains J, Penzien D (2006). The neuropsychology of recurrent headache. Headache.

[21] Kruit M, van Buchem M, Launer L, Terwindt G, Ferrari M (2010). Migraine is associated with an increased risk of deep white matter
lesions, subclinical posterior circulation infarcts and brain iron accumulation:
the population-based MRI CAMERA study. Cephalalgia.

[22] Porter A, Gladstone J, Dodick D (2005). Migraine and white matter hyperintensities. Curr Pain Headache Rep.

[23] Akerman S, Goadsby P (2007). Dopamine and migraine: biology and clinical
implications. Cephalalgia.

[24] Camarda C, Monastero R, Pipia C, Recca D, Camarda R (2007). Interictal executive dysfunction in migraineurs without aura:
relationship with duration and intensity of attacks. Cephalalgia.

[25] Azuma T (2004). Working Memory and Perseveration in Verbal Fluency. Neuropsychology.

[26] Baldo J, Schwartz S, Wilkins D, Dronkers N (2006). Role of frontal versus temporal cortex in verbal fluency as revealed
by voxel-based lesion symptom mapping. J Int Neuropsychol Soc.

[27] Hooker W, Raskin N (1986). Neuropsychologic alterations in classic and common
migraine. Arch Neurol.

[28] Meyer J, Thornby J, Crawford K, Rauch G (2000). Reversible cognitive decline accompanies migraine and cluster
headaches. Headache.

